# Associations of intensity, duration, cumulative dose, and age at start of smoking, with thyroid cancer in Chinese males: A hospital-based case–control study in Zhejiang Province

**DOI:** 10.18332/tid/130350

**Published:** 2020-12-01

**Authors:** Meng Wang, Wei W. Gong, Feng Lu, Qing F. He, Ru Y. Hu, Jie M. Zhong, Min Yu

**Affiliations:** 1 Zhejiang Provincial Center for Disease Control and Prevention, Hangzhou, China

**Keywords:** thyroid cancer, smoking, case–control study

## Abstract

**INTRODUCTION:**

There has been considerable research on the association between smoking status and thyroid cancer risk in males, yet the findings are inconsistent. In this study, we investigated the associations of intensity, duration, cumulative dose, and age at start of smoking, with thyroid cancer in Chinese males.

**METHODS:**

From a 1:1 matched case–control study conducted between 2015 and 2017 in Zhejiang Province, China, 676 pairs of male subjects were included in the analysis. The associations between smoking characteristics and thyroid cancer were evaluated in logistic regression models by odds ratios (ORs) with 95% confidence intervals (CIs).

**RESULTS:**

Compared with never smokers, the former smokers were 0.096 times (95% CI: 0.012–0.778) less likely to have thyroid cancer. The significant inverse association was not observed in current smokers (OR=0.333; 95% CI: 0.084–1.322). Among both former and current smokers, higher smoking intensity (>10 cigarettes/day), duration (>15 years), and cumulative dose of smoking (>10 packyears) were significantly associated with reduced occurrence of thyroid cancer.

**CONCLUSIONS:**

Our findings indicate that former smoking is inversely associated with thyroid cancer occurrence in Chinese males. The reduction in the occurrence of thyroid cancer was also confirmed for both former and current smokers with higher smoking intensity, duration, and cumulative dose.

## INTRODUCTION

Thyroid cancer incidence has been rising substantially worldwide. From 1990 to 2013, the global age-standardized incidence rate of thyroid cancer increased by 20%^1^. The overall thyroid cancer incidence in the United States increased 3% annually during 1974 and 2013^2^. In parallel, the incidence of thyroid cancer in South Korea rose by 14 times from 1993 to 2011^3^. Consistently, a significant trend of increasing incidence of thyroid cancer has been found in China in the past decades. Between 2000 and 2011, a dramatic rise in thyroid cancer incidence was observed among the female population in China, with an annual percentage change (APC) of 20.1%^4^. Meanwhile, the growing trend was confirmed in local Chinese populations in Shanghai, Hong Kong, Taiwan and Zhejiang^5-7^. According to previous studies, the markedly increasing incidence of thyroid cancer could be explained not only by the improved diagnostic techniques, but also by the elevated prevalence of individual risk factors (e.g. obesity) and exposure to environmental risk factors (e.g. iodine levels)^8,9^.

Tobacco use is the leading preventable cause of cancer worldwide. The World Health Organization (WHO) reported that at least 19 other cancer sites or sub-sites were designated as causally related to smoking^10^. However, a series of recent pooled analyses with case–control or cohort studies suggested reduced thyroid cancer risk in ever and current smokers, compared with never smokers^11,12^. Except for the smoking status, increasing studies have further revealed that the reduced risk was more pronounced in smokers with greater smoking intensity, duration and pack-years^13,14^. Consistent with these findings, mechanistic studies also provided biological plausibility in explaining the possible protective effect of smoking on thyroid cancer. It was widely assumed that the inverse association with thyroid cancer risk was due to lower levels of thyroid stimulating hormone (TSH), thyroid auto-antibodies and the anti-estrogenic effect in smokers^15-19^. Even so, given the considerable types of smoking-related cancers, further studies in this field are warranted.

With 315 million smokers, China is the largest producer and consumer of tobacco^20^. However, relevant evidence on the role of smoking in thyroid cancer occurrence among the Chinese population is lacking. To fill this knowledge gap, we carried out a hospital-based case–control study in Zhejiang Province to investigate the possible associations between intensity, duration, cumulative dose, and age at start of smoking, with thyroid cancer in Chinese males.

## METHODS

### Study subjects

To explore the associated factors of thyroid cancer, a 1:1 matched hospital-based case–control study was performed in 7 counties (Lucheng, Pingyang, Cangnan, Cixi, Nanhu, Changxing and Yongkang) of Zhejiang Province. Case subjects were eligible if they were first primary thyroid cancer diagnosed in local hospitals from July 2015 to December 2017. All the thyroid cancer cases were identified by physician review of medical records and pathology reports. Notably, among the cases, those who had a history of any cancer or were unable/unwilling to complete the study questionnaire were excluded. In the current study, thyroid cancer was further identified based on ICD-10 and referred to malignant neoplasm of thyroid gland (C73). In the same area and same year, control subjects were selected from thyroid-healthy examinees who underwent thyroid function tests and thyroid ultrasound screening in the annual routine physical examination, which was conducted in local hospitals. In Zhejiang Province, authorized by the government, fixed local hospitals organize physical examination covering all the insured residents in community or village once a year. We excluded subjects with abnormal levels of free triiodothyronine, free thyroxine, and TSH, respectively. Besides, we excluded those whose ultrasound findings showed suspected malignancy. Control subjects who had a history of any cancer or were unable/unwilling to complete the study questionnaire were also excluded. Finally, case subjects were matched to control subjects by age (plus or minus three years) and sex.

### Estimation of the sample size

In our study, the cases and controls were selected by 1:1, so we calculated the sample size from the following formulae:

$$M=\frac{m}{p_{0}q_{1}+p_{1}q_{0}}$$

m=[Uα/2+Uβ(p(1−p))]2/(p−1/2)2

$$p=\frac{OR}{1+OR}≅\frac{RR}{1+RR}$$

With

$$p_{1}=\frac{p_{0}OR}{1+p_{0}(OR-1)}$$

And

$$q_{1}=1-p_{1},q_{0}=1-p_{0}$$

where *p*_0_ is the estimated diabetes exposure rate in the control group, *p*_1_ is the estimated diabetes exposure rate in the case group, with values p_0_ = 0.07, *OR* = 2, *α* = 0.05, *β* = 0.10, so that the sample size *M* = 398 in each county, and actually 2937 pairs of subjects were recruited in 7 counties.

### Questionnaire

With a designed questionnaire, the interviewer collected all relevant information face-to-face at enrollment. The questionnaire was completed within two months after each pair of case and control was successfully matched. The questionnaire design went through literature review, panel discussion, check and approval by experts and revision after pilot study. The same structured questionnaire was administered to each pair of subjects by the same trained interviewer. The questionnaire was used to collect information on sociodemographic characteristics, individual history and family history of chronic diseases, lifestyle behaviors, environmental hazardous substances exposure, and dietary habits, etc. The specific content of the questionnaire has been described in detail elsewhere^21^, and is thus only briefly described here. This study was carried out in accordance with the Declaration of Helsinki 1975, and verbal consents were obtained from participants and approved by the Ethics Committee of Zhejiang Provincial Center for Disease Control and Prevention (CDC). The ethics committee approved the procedure for verbal consent because Zhejiang CDC has the authority of the Zhejiang provincial government to collect and publish the cancer related information, which is part of disease surveillance scope in Zhejiang CDC. All the participants (cases and controls) were notified that they have the right to refuse or terminate the study at any point of the interview. Because we obtained verbal consent, documentation of consent was not required. However, the information provided by each participant was kept confidential in Zhejiang CDC.

### Definition of smoking variables

Information on smoking status, intensity, duration, and age at start of smoking, was collected via the designed questionnaire at enrollment. Smoking status was grouped as never, former, and current. Former smokers were defined as subjects who have quitted smoking at least 1 year. Current smokers were defined as subjects who smoked currently or daily. Smoking intensity was defined as the average number of cigarettes smoked per day and dichotomized as ≤10 and >10 cigarettes. Duration of smoking was defined as the total smoking years reported by the former and current smokers and dichotomized as ≤15 and >15 years. The cumulative dose of smoking was calculated as pack-years (the number of cigarettes smoked per day divided by 20 [assuming 20 cigarettes per pack] and multiplied by the duration of smoking years). We dichotomized pack-years as ≤10 and >10. The variable, age at start of smoking (dichotomized as ≤20 and >20 years) was also included in the analysis. All smoking variables were grouped to be as consistent, as possible, with previously published studies on this topic, based on the sample sizes in each group.

### Definition of the covariates

The following variables were collected at enrollment and chosen as the potential confounders. Age (≤40 and >40 years), marriage status (unmarried, married), education level (no formal/primary school, middle/high school, college/university/postgraduate), average monthly household income (≤2000; 2000–5000; >5000 RMB; with 1000 Chinese Renminbi about 150 US$), alcohol drinking (never, occasional, current regular), body mass index (underweight, normal weight, overweight, obese), family history of thyroid cancer (negative, positive) were included in the analyses. The body mass index (BMI: kg/m^2^) was calculated as weight divided by the square of height. According to the Chinese adult BMI classification proposed by the Working Group on Obesity in China in 2001^22^, subjects were categorized as underweight (<18.5), normal weight (18.5–23.9), overweight (24.0–27.9), or obese (≥28.0).

### Statistical analysis

Descriptive statistics were used to describe the baseline characteristics of male subjects with frequency (n) and proportion (%). Univariate conditional logistic regression models were performed to examine the relationships of the covariates with the thyroid cancer.

To examine the associations between smoking status, intensity, duration, cumulative dose, and age at start of smoking, with thyroid cancer, we conducted four multivariable conditional logistic regression models to adjust for the covariates and the possible effects, by calculating odds ratios (ORs) and 95% confidence intervals (CIs). The intensity, duration, cumulative dose, and age at start of smoking, were analyzed in combination with smoking status for former and current smokers. In Model 1, only age was adjusted. In Model 2, age and other sociodemographic characteristics including education level, average monthly household income, marriage status, and family history of thyroid cancer were included. Model 3 adjusted as for Model 2 plus the health behavior of alcohol intake. Model 4 adjusted as for Model 3 plus BMI. Subgroup analyses were performed stratified by age (≤40 and >40 years), BMI (normal/underweight, obese/overweight), and alcohol intake (non-drinker, drinker) in multivariable logistic regression models; only the final results (Model 4) are presented. All analyses were based on the two-sided 5% level of significance and performed using SAS statistical package (version 9.2, SAS Institute, Inc., Cary, NC, USA).

## RESULTS

### Baseline characteristics of subjects

A total of 2937 pairs of subjects participated in the case–control study. Among them, there were 2261 pairs (77.0%) of females and 676 pairs (23.0%) of males. Considering the extremely low prevalence of smoking in females (former and current, 0.42%), we only included the male subjects in the present analysis. The mean (SD) age in male case and control subjects was 48.71 (1.29) years and 48.61 (1.29) years, respectively. Of the 1352 males, there were 175 former smokers (12.94%) and 587 current smokers (43.42%). Frequencies and proportions of baseline characteristics among case and control subjects are shown in Table 1. Compared to control subjects, case subjects were more likely to have higher education (college/university/postgraduate, p=0.016), be overweight (p<0.001) or obese (p=0.041), and less likely to drink alcohol occasionally (p=0.001) and current regularly (p<0.001).

**Table 1:** The baseline characteristics of male subjects and their relationships with thyroid cancer

*Factors*	*Cases (N=676) n (%)*	*Controls (N=676) n (%)*	*p*	*OR*	*95 % CI*
**Age** (years), mean±SD	48.71±1.29	48.61±1.29			
>40	489 (72.34)	491 (72.63)			
≤40	187 (27.66)	185 (27.37)			
**Education level**					
No formal/primary school	169 (25.00)	178 (26.33)		Ref.	
Middle/high school	318 (47.04)	346 (51.18)	0.672	1.073	0.774–1.488
College/university/postgraduate	187 (27.66)	150 (22.19)	0.016	1.701	1.105–2.619
**Marriage status**					
Unmarried	50 (8.14)	49 (7.25)		Ref.	
Married	621 (91.86)	626 (92.60)	0.893	0.964	0.568–1.636
**Average monthly household income** (RMB)					
≤2000	481 (71.15)	462 (68.34)		Ref.	
2000–5000	72 (10.65)	81 (11.99)	0.208	0.776	0.523–1.152
>5000	123 (18.20)	133 (19.67)	0.201	0.786	0.544–1.137
**Body mass index**					
Normal weight	296 (43.79)	377 (55.77)		Ref.	
Underweight	12 (1.78)	19 (2.81)	0.599	0.813	0.376–1.758
Overweight	277 (40.98)	223 (32.99)	<0.001	1.704	1.324–2.193
Obese	66 (9.76)	56 (8.28)	0.041	1.523	1.018–2.279
**Family history of thyroid cancer**					
Negative	17 (2.51)	3 (0.44)		Ref.	
Positive	127 (18.79)	106 (15.68)	0.341	3.000	0.312–28.841
**Alcohol drinking**					
Never	331 (48.96)	237 (35.06)		Ref.	
Occasional	205 (30.33)	221 (32.69)	0.001	0.624	0.470–0.828
Current regular	140 (20.71)	218 (32.25)	<0.001	0.417	0.309–0.562

OR: odds ratio. CI: confidence interval. Ref.: reference. Percentages of each variable may not add up to 100% because of missing data or rounding off. RMB: 1000 Chinese Renminbi about 150 US$.

### The associations between smoking characteristics and thyroid cancer

Table 2 shows the results of multivariable models investigating the associations between smoking status, intensity, duration, cumulative dose, and age at start of smoking, with thyroid cancer (see also Supplementary file). In Model 1, compared with never smokers, the former smokers were 1.619 times (95% CI: 1.115–2.351) more likely to have thyroid cancer, after adjusting for age. The consistent effects were also found among former smokers with higher intensity (>10 cigarettes/day), shorter duration (≤15 years), lower cumulative dose of smoking (≤10 packyears) and later age at start of smoking (>20 years), with an OR of 1.717 (95% CI: 1.085–2.716), 1.986 (95% CI: 1.105–3.571), 1.762 (95% CI: 1.014–3.061), and 1.748 (95% CI: 1.075–2.844), respectively. With further adjustment for other sociodemographic characteristics and health behavior of alcohol in Models 2 and 3, these effects of smoking on thyroid cancer were largely inversed and attenuated to zero. However, after adjustment for the BMI in Model 4, a reduced occurrence of thyroid cancer was observed in former and current smokers with different smoking characteristics. Compared with never smokers, the former smokers were 0.096 times (95% CI: 0.012–0.778) less likely to have thyroid cancer. Consistent in both former and current smokers, higher intensity (>10 cigarettes/day), higher duration (>15 years), and higher cumulative dose of smoking (>10 packyears) were significantly associated with reduced occurrence of thyroid cancer. Subgroup analyses showed that the associations between smoking status, intensity, duration, cumulative dose, age at start of smoking, and thyroid cancer were modified by age, BMI and alcohol intake, although no interactions were observed with any of the variables.

**Table 2:** Multivariate analyses – associations between smoking status, as well as intensity, duration, cumulative dose and age at start of smoking with thyroid cancer in males

*Factors*	*Cases (N=676)*	*Controls (N=676)*	*Model 1*		*Model 2*		*Model 3*		*Model 4*	
			*OR^†^*	*95% CI*	*OR^†^*	*95% CI*	*OR^†^*	*95% CI*	*OR^†^*	*95 % CI*
**Smoking status**										
Never smoker	294	296	Ref.		Ref.		Ref.		Ref.	
Former smoker	106	69	**1.619**	**1.115–2.351**	0.214	0.044–1.042	0.251	0.047–1.342	**0.096**	**0.012–0.778**
Current smoker	276	311	0.890	0.692–1.145	0.571	0.186–1.749	0.622	0.194–1.996	0.333	0.084–1.322
**Intensity of smoking** (cigarettes/day)										
Never smoker	294	296	Ref.		Ref.		Ref.		Ref.	
≤10 FS	41	29	1.471	0.865–2.503	0.163	0.019–1.407	0.143	0.014–1.476	0.086	0.006–1.228
>10 FS	65	40	**1.717**	**1.085–2.716**	0.160	0.021–1.248	0.182	0.019–1.727	**0.045**	**0.003–0.802**
≤10 CS	112	123	0.917	0.671–1.254	1.129	0.296–4.306	1.227	0.307–4.897	0.601	0.110–3.296
>10 CS	164	188	0.866	0.640–1.172	0.215	0.043–1.071	0.230	0.044–1.203	**0.131**	**0.020–0.847**
**Duration of smoking** (years)										
Never smoker	294	296	Ref.		Ref.		Ref.		Ref.	
≤15 FS	36	18	**1.986**	**1.105–3.571**	0.445	0.063–3.139	0.495	0.059–4.160	0.132	0.007–2.358
>15 FS	70	51	1.487	0.960–2.302	**0.064**	**0.005–0.791**	0.089	0.007–1.151	**0.013**	**0.001–0.378**
≤15 CS	58	71	0.798	0.531–1.200	1.161	0.140–9.650	1.176	0.138–9.995	1.590	0.154–16.379
>15 CS	218	240	0.928	0.700–1.232	0.437	0.114–1.679	0.475	0.120–1.886	**0.129**	**0.018–0.917**
**Cumulative dose of smoking** (packyears)										
Never smoker	294	296	Ref.		Ref.		Ref.		Ref.	
≤10 FS	39	22	**1.762**	**1.014–3.061**	0.211	0.025–1.775	0.228	0.024–2.159	0.097	0.007–1.444
>10 FS	67	47	1.491	0.950–2.340	0.158	0.020–1.282	0.188	0.021–1.726	**0.044**	**0.002–0.784**
≤10 CS	92	92	0.981	0.697–1.381	2.531	0.365–17.529	2.333	0.325–16.755	1.386	0.161–11.928
>10 CS	184	219	0.835	0.625–1.115	0.337	0.090–1.269	0.367	0.093–1.446	**0.172**	**0.033–0.892**
**Age started smoking** (years)										
Never smoker	294	296	Ref.		Ref.		Ref.		Ref.	
≤20 FS	54	39	1.497	0.919–2.439	0.217	0.031–1.514	0.239	0.031–1.850	0.096	0.008–1.111
>20 FS	52	30	**1.748**	**1.075–2.844**	0.216	0.029–1.585	0.269	0.034–2.133	0.100	0.008–1.209
≤20 CS	145	163	0.894	0.661–1.210	0.634	0.192–2.086	0.699	0.204–2.388	0.351	0.080–1.529
>20 CS	131	148	0.885	0.648–1.209	0.441	0.098–1.987	0.468	0.097–2.262	0.302	0.053–1.724

FS: former smoker. CS: current smoker. OR: odds ratio. CI: confidence interval. Ref.: reference. Bold numbers represent significant results.

† Model 1 only adjusted for age; Model 2 adjusted as for Model 1 plus sociodemographic characteristics including education level, average monthly household income, marriage status, and family history of thyroid cancer; Model 3 adjusted as for Model 2 plus the health behavior of alcohol intake; Model 4 adjusted as for Model 3 plus BMI.

## DISCUSSION

Overall, findings from this study indicate an inverse association between former versus never smoking and thyroid cancer occurrence in Chinese males, while this was not statistically significant in current smokers after adjustment for potential confounders. In combination with different measures of smoking characteristics, our results further confirmed that higher smoking intensity, duration, and cumulative dose were significantly associated with reduced thyroid cancer occurrence among both former and current smokers. However, when stratified by age, BMI and alcohol intake, these observed associations were attenuated to zero. The present study is probably one of the few studies attempting to investigate the role of smoking in thyroid cancer occurrence in the Chinese population.

There has been considerable research on the association between smoking status and thyroid cancer risk in males, yet the findings are inconsistent. Specifically, a pooled analysis of 14 case–control studies conducted in 2003 indicated that former smoking was associated with decreased thyroid cancer risk, while the effect was not pronounced (OR=0.9; 95% CI: 0.6–1.3)^14^. The lower risk of thyroid cancer among former smokers was confirmed in a recent cohort study, and similarly, this association did not reach statistical significance (HR=0.90; 95% CI: 0.71–1.14)^23^. In addition, another pooled analysis of five prospective studies in the United States reported a non-significant positive association between former smoking and thyroid cancer risk (OR=1.10; 95% CI: 0.86–1.40)^13^. In our study, although findings were consistent with the literature, where studies have reported reduced risk associated with former smoking, it is noteworthy that our observed effect was statistically significant (OR=0.096; 95% CI: 0.012–0.778). Regarding current smoking, we showed a non-significant inverse association with thyroid cancer in males (OR=0.333; 95% CI: 0.084–1.322), which was in accord with findings from two independent pooled analysis of case–control (OR=0.7; 95% CI: 0.5–1.0) and prospective studies (OR=0.83; 95% CI: 0.55–1.24)^13,14^. The possible decreased risk of thyroid cancer in current smokers was further confirmed in a large cohort study where a significant association was reported (HR=0.56; 95% CI: 0.43–0.72)^23^. Besides, a non-significant positive association between current smoking and thyroid cancer (OR=1.36; 95% CI: 0.44–4.26) was also found in a countrywide case–control study in New Caledonia^24^. Similar to the smoking status, the relationships between other smoking characteristics and thyroid cancer risk seemed to be inconclusive as well. In our study, the results showed that higher smoking intensity, duration, and cumulative dose were significantly associated with reduced thyroid cancer occurrence in males, which were well confirmed in a Canadian case–control study^25^. In another case–control study in the United States, greater smoking intensity but not duration was found to be associated with a lower risk of thyroid cancer^26^. Our finding on the cumulative dose of smoking was also consistent with result from a recent Korean cohort study, where a dose-dependent inverse association between smoking pack-years and thyroid cancer risk was reported^23^. Besides, according to a case–control study in Serbia, the authors found that neither smoking intensity nor duration was significantly associated with thyroid cancer in men^27^. In theory, the varied effects on thyroid cancer risk reported by these studies could be due to the differences in sample size, study design, population characteristics, and measures of smoking exposures. In addition, when stratified by age, BMI and alcohol intake, we found that the significant associations between smoking characteristics and thyroid cancer were attenuated to zero, which may be due to the small number of cases in each subgroup.

Possible biological mechanisms linking the reduced risk of thyroid cancer and smoking have been studied and hypothesized to rely on the low levels of TSH, thyroid auto-antibodies, and anti-estrogenic effect in smokers^15-19^. Other chemical constituents of cigarettes such as nicotine and anatabine may also play a contributory role^28,29^, which requires further confirmation in mechanistic studies. Besides, we speculated that the difference in the risk of thyroid cancer between former and current smokers was due to the ‘sick-quitter effect’. Specifically, the former smokers might be inspired to abstain due to health problems such as diabetes; based on prior knowledge^30,31^, the risk of thyroid cancer in diabetes patients is much lower, possibly due to the effects of diabetes drugs (e.g. metformin).

### Strengths and limitations

Our study had several strengths. This is one of the few studies investigating the role of smoking in thyroid cancer occurrence among the Chinese population. In the hospital-based case–control study, all incident thyroid cancer cases were diagnosed in hospitals and further identified by physician review of medical records and pathology reports, which would minimize the probability of misclassification.

However, some limitations were also observed. First, only males were included in the study and the relatively small number of subjects could limit the statistical power to investigate the associations between smoking characteristics and thyroid cancer. Second, with a retrospective design and self-reported smoking characteristics, recall bias may be inevitable in this study. Third, the information of years since quitting smoking was not collected at baseline, limiting our ability to evaluate the effect of former smoking on thyroid cancer.

## CONCLUSIONS

Findings from the present case–control study indicate that former smoking is inversely associated with thyroid cancer occurrence in Chinese males. The reductions in occurrence were also confirmed in both former and current smokers with higher smoking intensity, duration, and cumulative dose.
