# Supplementary material for: Associations of intensity, duration, cumulative dose, and age at start of smoking, with thyroid cancer in Chinese males: A hospital-based case–control study in Zhejiang Province
Source: Tob Induc Dis. 2020 Dec 1;18:97. doi: 10.18332/tid/130350 (PMC7713696; doi:10.18332/tid/130350)
Supplement: Supplementary file 1 [file TID-18-97-s1.pdf]

Supplementary Table S1. Stratified analyses by age-Associations between smoking status, as well as intensity, duration, cumulative dose and age at start of smoking with thyroid cancer in males.

Factors	Overall		Age ≦ 40 years		Age >40 years		P value for interaction[Δ]
	OR[†]	95%CI	OR[†]	95%CI	OR[†]	95%CI	
Smoking status							0.219
Never smoker	Ref.		Ref.		Ref.		
Former smoker	**0.096**	**0.012-0.778**	NA	NA	0.901	0.344-2.364	
Current smoker	0.333	0.084-1.322	0.741	0.170-3.234	0.846	0.406-1.761	
Intensity of smoking (cigarettes/day)							0.986
Never smokers	Ref.		Ref.		Ref.		
Former smoker+ ≦10	0.086	0.006-1.228	NA	NA	1.573	0.339-7.299	
Former smoker+ >10	**0.045**	**0.003-0.802**	NA	NA	0.668	0.213-2.100	
Current smoker+ ≦10	0.601	0.110-3.296	2.067	0.302-14.152	0.723	0.249-2.099	
Current smoker+ >10	**0.131**	**0.020-0.847**	0.063	0.004-1.037	0.863	0.398-1.873	
Duration of smoking (years)							0.997
Never smokers	Ref.		Ref.		Ref.		
Former smoker+ ≦15	0.132	0.007-2.358	NA	NA	1.789	0.382-8.371	
Former smoker+ >15	**0.013**	**0.001-0.378**	NA	NA	0.636	0.206-1.963	
Current smoker+ ≦15	1.590	0.154-16.379	0.921	0.190-4.459	0.637	0.131-3.104	
Current smoker+ >15	**0.129**	**0.018-0.917**	0.312	0.024-4.062	0.855	0.406-1.802	
Cumulative dose of smoking (pack-years)							0.989
Never smokers	Ref.		Ref.		Ref.		
Former smoker+ ≦10	0.097	0.007-1.444	NA	NA	1.443	0.295-7.050	
Former smoker+ >10	**0.044**	**0.002-0.784**	NA	NA	0.726	0.238-2.211	
Current smoker+ ≦10	1.386	0.161-11.928	2.213	0.332-14.777	0.953	0.293-3.093	
Current smoker+ >10	**0.172**	**0.033-0.892**	**0.038**	**0.002-0.644**	0.805	0.373-17.33	
Age started smoking (years)							0.540
Never smokers	Ref.		Ref.		Ref.		
Former smoker+ ≦20	0.096	0.008-1.111	NA	NA	0.575	0.144-2.292	
Former smoker+ >20	0.100	0.008-1.209	NA	NA	1.196	0.376-3.808	
Current smoker+ ≦20	0.351	0.080-1.529	0.919	0.157-5.363	0.876	0.398-1.930	
Current smoker+ >20	0.302	0.053-1.724	0.561	0.084-3.747	0.751	0.292-1.934	

OR odds ratio, CI confidence interval, Ref. reference, NA No available due to the limited number of subjects. Bold numbers represent significant results.

† Model 4: adjusted for age, education level, average monthly household income, marriage status, family history of thyroid cancer, alcohol intake and BMI.

Δ Effect modification was tested by adding interaction terms between these smoking factors and age independently to the model.

Supplementary Table S2. Stratified analyses by BMI-Associations between smoking status, as well as intensity, duration, cumulative dose and age at start of smoking with thyroid cancer in males.

Factors	Overall		Under/normal weight		Overweight/obese		P value for interaction$^{\Delta}$
	OR†	95%CI	OR†	95%CI	OR†	95%CI	
Smoking status							0.709
Never smoker	Ref.		Ref.		Ref.		
Former smoker	**0.096**	**0.012-0.778**	1.492	0.434-5.127	0.393	0.100-1.545	
Current smoker	0.333	0.084-1.322	0.870	0.372-2.034	0.760	0.288-2.007	
Intensity of smoking (cigarettes/day)							1.000
Never smokers	Ref.		Ref.		Ref.		
Former smoker+ ≦10	0.086	0.006-1.228	0.756	0.141-4.057	1.725	0.142-20.962	
Former smoker+ >10	**0.045**	**0.003-0.802**	2.708	0.457-16.061	0.186	0.031-1.134	
Current smoker+ ≦10	0.601	0.110-3.296	1.622	0.446-5.902	0.825	0.242-2.816	
Current smoker+ >10	**0.131**	**0.020-0.847**	0.716	0.282-1.822	0.686	0.234-2.013	
Duration of smoking (years)							0.998
Never smokers	Ref.		Ref.		Ref.		
Former smoker+ ≦15	0.132	0.007-2.358	1.042	0.195-5.583	0.937	0.110-7.982	
Former smoker+ >15	**0.013**	**0.001-0.378**	1.792	0.354-9.075	0.231	0.041-1.293	
Current smoker+ ≦15	1.590	0.154-16.379	1.714	0.432-6.797	0.953	0.197-4.610	
Current smoker+ >15	**0.129**	**0.018-0.917**	0.675	0.260-1.750	0.664	0.227-1.941	
Cumulative dose of smoking (pack-years)							0.995
Never smokers	Ref.		Ref.		Ref.		
Former smoker+ ≦10	0.097	0.007-1.444	0.751	0.119-4.755	1.064	0.133-8.508	
Former smoker+ >10	**0.044**	**0.002-0.784**	1.936	0.388-9.660	0.203	0.033-1.259	
Current smoker+ ≦10	1.386	0.161-11.928	4.484	0.991-20.281	0.716	0.184-2.787	
Current smoker+ >10	**0.172**	**0.033-0.892**	0.485	0.184-1.278	0.745	0.253-2.193	
Age started smoking (years)							0.969
Never smokers	Ref.		Ref.		Ref.		
Former smoker+ ≦20	0.096	0.008-1.111	0.949	0.165-5.450	0.252	0.035-1.799	
Former smoker+ >20	0.100	0.008-1.209	2.166	0.431-10.892	0.527	0.100-2.766	
Current smoker+ ≦20	0.351	0.080-1.529	0.950	0.376-2.399	0.805	0.274-2.370	
Current smoker+ >20	0.302	0.053-1.724	0.741	0.238-2.300	0.686	0.206-2.280	

OR odds ratio, CI confidence interval, Ref. reference. Bold numbers represent significant results.

† Model 4 adjusted for age, education level, average monthly household income, marriage status, family history of thyroid cancer, alcohol intake and BMI.

$^{\Delta}$ Effect modification was tested by adding interaction terms between these smoking factors and BMI independently to the model.

Supplementary Table S3. Stratified analyses by alcohol intake-Associations between smoking status, as well as intensity, duration, cumulative dose and age at start of smoking with thyroid cancer in males.

Factors	Overall		Non-drinker		Drinker		P value for interaction$^{\Delta}$
	OR†	95%CI	OR†	95%CI	OR†	95%CI	
Smoking status							0.510
Never smoker	Ref.		Ref.		Ref.		
Former smoker	**0.096**	**0.012-0.778**	1.094	0.213-5.624	0.514	0.153-1.727	
Current smoker	0.333	0.084-1.322	0.446	0.164-1.213	1.128	0.481-2.647	
Intensity of smoking (cigarettes/day)							0.940
Never smokers	Ref.		Ref.		Ref.		
Former smoker+ ≦10	0.086	0.006-1.228	0.582	0.062-5.428	0.926	0.148-5.778	
Former smoker+ >10	**0.045**	**0.003-0.802**	1.979	0.184-21.225	0.338	0.073-1.568	
Current smoker+ ≦10	0.601	0.110-3.296	0.306	0.076-1.224	1.466	0.465-4.621	
Current smoker+ >10	**0.131**	**0.020-0.847**	0.550	0.182-1.658	0.964	0.373-2.490	
Duration of smoking (years)							0.882
Never smokers	Ref.		Ref.		Ref.		
Former smoker+ ≦15	0.132	0.007-2.358	0.507	0.051-5.019	1.787	0.309-10.336	
Former smoker+ >15	**0.013**	**0.001-0.378**	2.004	0.188-21.344	0.234	0.048-1.133	
Current smoker+ ≦15	1.590	0.154-16.379	0.978	0.134-7.137	1.069	0.315-3.620	
Current smoker+ >15	**0.129**	**0.018-0.917**	0.372	0.121-1.138	1.128	0.435-2.924	
Cumulative dose of smoking (pack-years)							0.978
Never smokers	Ref.		Ref.		Ref.		
Former smoker+ ≦10	0.097	0.007-1.444	0.597	0.066-5.381	1.035	0.156-6.859	
Former smoker+ >10	**0.044**	**0.002-0.784**	1.898	0.178-20.225	0.322	0.075-1.378	
Current smoker+ ≦10	1.386	0.161-11.928	0.619	0.144-2.655	1.962	0.560-6.871	
Current smoker+ >10	**0.172**	**0.033-0.892**	0.396	0.130-1.202	0.854	0.332-2.198	
Age started smoking (years)							0.906
Never smokers	Ref.		Ref.		Ref.		
Former smoker+ ≦20	0.096	0.008-1.111	3.528	0.199-62.556	0.144	0.018-1.154	
Former smoker+ >20	0.100	0.008-1.209	0.512	0.072-3.645	1.008	0.242-4.208	
Current smoker+ ≦20	0.351	0.080-1.529	0.449	0.141-1.424	1.154	0.463-2.874	
Current smoker+ >20	0.302	0.053-1.724	0.389	0.109-1.388	0.949	0.305-2.948	

OR odds ratio, CI confidence interval, Ref. reference. Bold numbers represent significant results.

† Model 4 adjusted for age, education level, average monthly household income, marriage status, family history of thyroid cancer, alcohol intake and BMI.

$^{\Delta}$ Effect modification was tested by adding interaction terms between these smoking factors and alcohol intake independently to the model.

© 2020 Wang M. et al.
